# CCR3 and Choroidal Neovascularization

**DOI:** 10.1371/journal.pone.0017106

**Published:** 2011-02-15

**Authors:** Yiwen Li, Deqiang Huang, Xin Xia, Zhengying Wang, Lingyu Luo, Rong Wen

**Affiliations:** 1 Bascom Palmer Eye Institute, University of Miami Miller School of Medicine, Miami, Florida, United States of America; 2 Neuroscience Program, University of Miami Miller School of Medicine, Miami, Florida, United States of America; Brigham and Women's Hospital, United States of America

## Abstract

Age-related macular degeneration (AMD) is the leading cause of irreversible blindness in the elderly in industrialized countries. The “wet” AMD, characterized by the development of choroidal neovacularization (CNV), could result in rapid and severe loss of central vision. The critical role of vascular endothelial growth factor A (VEGF-A) in CNV development has been established and VEGF-A neutralization has become the standard care for wet AMD. Recently, CCR3 was reported to play an important role in CNV development and that CCR3 targeting was reported to be superior to VEGF-A targeting in CNV suppression. We investigated the role of CCR3 in CNV development using the Matrigel induced CNV and found that in both rats and mice, CNV was well-developed in the control eyes as well as in eyes treated with CCR3 antagonist SB328437 or CCR3 neutralizing antibodies. No statistically significant difference in CNV areas was found between the control and SB328437 or CCR3-ab treated eyes. Immunostaining showed no specific expression of CCR3 in or near CNV. In contrast, both VEGF-A neutralizing antibodies and rapamycin significantly suppressed CNV. These results indicate that CCR3 plays no significant role in CNV development and question the therapeutic approach of CCR3 targeting to suppress CNV. On the other hand, our data support the therapeutic strategies of VEGF-A and mTOR (mammalian target of rapamycin) targeting for CNV.

## Introduction

Age-related macular degeneration (AMD) is the leading cause of blindness in the elderly in the developed world [1]. AMD presents in two distinct forms: the geographic atrophy and the exudative AMD. The geographic atrophy, also known as the “dry” form of AMD, is characterized by atrophy of the central retina. The exudative or “wet” AMD, a major cause of severe vision loss, is identified by the presence of choroidal neovascularization (CNV), new blood vessels originated from the choroid that invade the macula area. Development of CNV leads to retinal edema and may eventually destroy the structure of the retina, resulting in irreversible loss of central vision due to hemorrhage, retinal detachment and disciform scar formation.

Compiling evidence indicates clearly that VEGF-A, the major regulator of vasculogenesis and angiogenesis [2], plays a critical role in CNV development [3], [4], [5]. In animal CNV models, blocking VEGF-A by pharmacological agents not only effectively inhibits CNV [6], [7], but also induces regression of the newly developed CNV [8]. Clinically, neutralization of VEGF-A has become the standard care for wet AMD [9], [10], [11], [12], [13], [14].

CCR3 is a G protein coupled receptor that expressed mainly in eosinophils, basophils, a subset of T_h_2 lymphocyts, and mast cells, with the highest levels in eosinophils [15], [16], [17]. It binds to several CC ligands and is believed to function in recruiting leukocytes, mainly the T_h_2 cells and eosinophils, to inflammatory sites, and in allergic asthma, atopic dermatitis, and allergic rhinitis [15], [17], [18]. It is also found in vascular endothelial cells, including human microvascular endothelial cells, and has been shown to be involved in angiogenesis [19]. Recently, Takeda and colleagues reported that CCR3 played a critical role in CNV development [20]. They showed that CCR3 was specifically expressed in choroidal neovascular endothelial cells in tissues from human AMD patients. They also demonstrated that blocking CCR3 suppressed new vessel formation both in cultured human choroidal endothelial cells and in laser-induced CNV in mouse. In addition, their data indicated that CCR3 targeting was superior to VEGF-A neutralization in CNV suppression [20]. These investigators thus believe that CCR3 is a target for AMD therapy [20].

We studied the role of CCR3 in CNV development in Matrigel CNV model in both rat and mouse. In the model, CNV is induced by subretinal injection of Matrigel, as described in detail recently [8]. Here we report that blocking CCR3 with either a small molecular antagonist SB328437 or CCR3 neutralizing antibodies (CCR3-ab) failed to inhibit CNV in both rat and mouse. On the other hand, VEGF-A neutralizing antibodies (VEGF-ab) efficaciously inhibited CNV development in the Matrigel model. Rapamycin, a known CNV inhibitor [21], was also very effective in suppressing CNV development. Our results therefore argue against the role of CCR3 in CNV development and question whether CCR3 targeting is a viable therapeutic approach for CNV.

## Results

### Development of CNV in the Matrigel model

**Figure 1:**
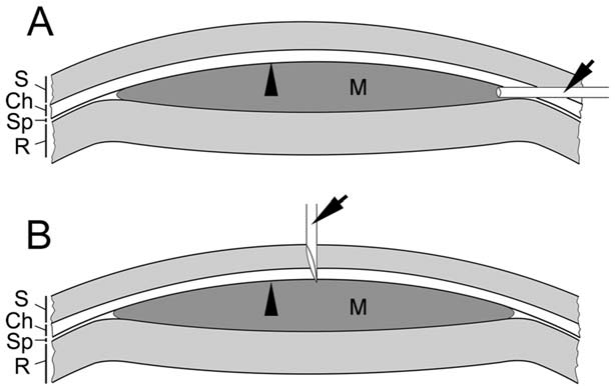
Schematic illustration of subretinal injection of Matrigel. The tip of a blunt needle (A, arrow) is introduced to the subretinal space (Sp) at a shallow angle toward the posterior pole. Injected Matrigel pushes its way to form a bleb in the subretinal space (M, dark shaded area). A sharp needle (B, arrow) is used to break the Bruch's membrane (A and B, arrowhead) in the center of the Matrigel bleb after Matrigel injection (B). S: sclera; Ch: choroid; R: retina, M: Matrigel.

**Figure 2:**
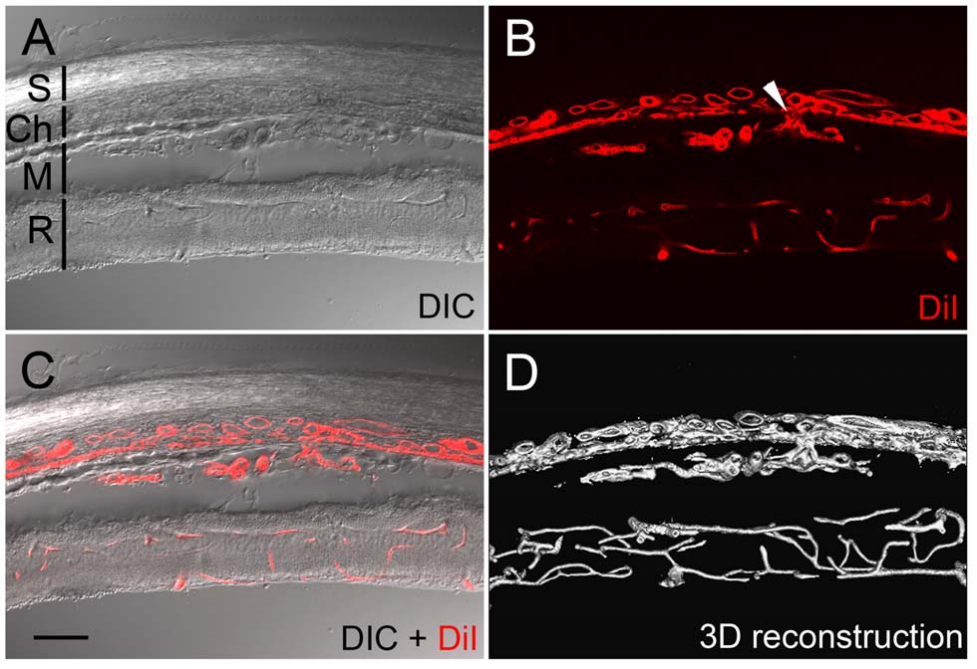
CNV induced by subretinal Matrigel. Eyes were collected 10 days after subretinal injection of Matrigel in Sprague-Dawley rats. A DIC image (A) shows injected Matrigel (M) and the layers of the eye, including the sclera (S), choroid (Ch), and the retina (R). CNV is clearly shown by DiI staining, along with choroidal and retina vasculature (B) The site where the CNV entered the Matrigel area is indicated by an arrowhead (B). A merged image of A and B is presented in C to show the structure of the eye related to the CNV. A 3D image of the vasculature in the entire thickness of the section was reconstructed in panel D. Scale bar: 100 µm.

In the Matrigel model, CNV was induced by injection of Matrigel into the subretinal space [8], [22] (Fig. 1, see Materials and Methods for details). Angiogenic sprouts are detected 4 days after injection. The CNV network is well developed 10 days after injection and increases progressive in size [8]. Figure 2 shows a typical CNV network in the Matrigel injected area. A DIC (differential interference contrast) image of this 100 µm-thick section displays the location of injected Matrigel related to the choroid and the retina (Fig. 2A). A fluorescent confocal image (Fig. 2B) shows the induced CNV along with the choroidal and retinal vasculature. The site indicated by a white arrow in Fig. 2B clearly shows that the neovasculature originated from choriocapillaris. The CNV network is better appreciated in Fig. 2C in which the two images in Fig. 2A and 2B were merged, and in Fig. 2D in which a 3-D image of the blood vessels in the entire thickness of the section was reconstructed.

### Hyperpermeability of newly developed CNV

**Figure 3:**
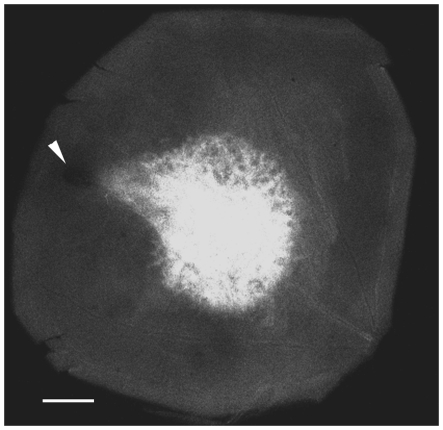
Hyperpermeability of CNV. Rats were injected with Matrigel to the subretinal space. Ten days later, Evans Blue (60 mg/kg, i.v.) was give for 30 min before eyes were harvested. Evans Blue fluorescence in the choroid-retina preparation is concentrated in the Matrigel injected area, indicating hyperpermeability of CNV. The needle site is indicated (arrowhead). Scale bar: 500 µm.

Newly formed blood vessels are highly permeable because of the influence of VEGF-A (also known as vascular permeability factor or VPF) [23], [24]. The permeability of the subretinal Matrigel-induced CNV was examined with Evans Blue [25], a dye that tightly binds to albumin. A representative image of a choroid-retina preparation is shown in Fig. 3. Evans Blue signal is concentrated in Matrigel injected area, demonstrating the hyperpermeability of Matrigel-induced CNV.

### CCR3 targeting and CNV development in rat

**Figure 4:**
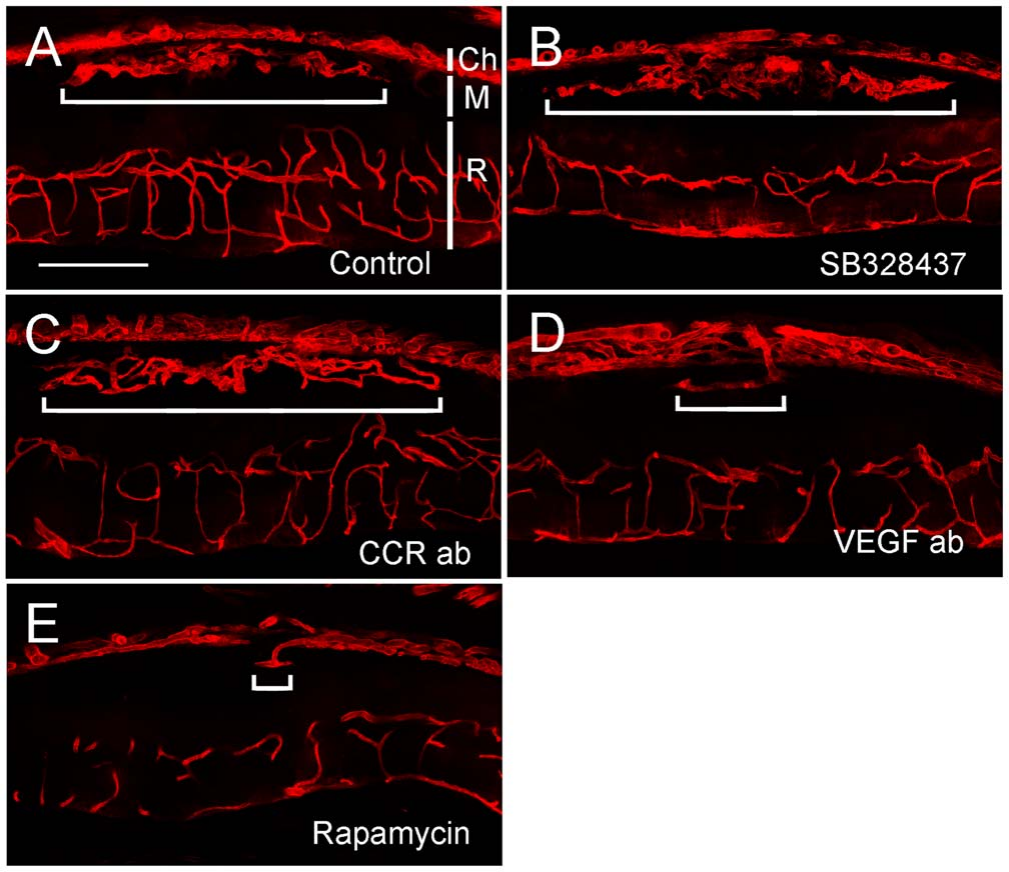
Effects of CCR3, VEGF-A, or mTOR inhibition on CNV in rats. Eyes were injected with Matrigel alone or mixed with SB328437, CCR3-ab, VEGF-ab, or rapamycin, and collected 12 days later. Extensive CNV was seen in the control eyes (A) as well as the eyes treated with SB328437 (B), CCR3-ab (C). In contrast, minimal CNV was found in eyes treated with VEGF-ab (D) or rapamycin (E). The width of CNV is indicated by a horizontal bar in each section. The layers of the eye, including the choroids (Ch), the Matrigel layer (M), and the retina (R), are indicated by vertical white bars in panel A. Scale bar: 200 µm.

To investigate whether CCR3 targeting blocks CNV development, we used a small molecule CCR3 antagonist SB328437 and specific CCR3-ab at the doses reported to inhibit CNV development by Takeda and colleagues that [20]. Surprisingly, no inhibition of CNV was seen in eyes treated with either SB328437 or CCR3-ab (Fig. 4A, 4B and 4C). In contrast, minimal CNV was seen in VEGF-ab treated eyes (Fig. 4D), and in eyes treated with rapamycin (Fig. 4E). In fact, CNV was completely absent in 10 out of 14 eyes treated with VEGF-A antibodies, and in 13 out of 16 eyes treated with rapamycin.

**Figure 5:**
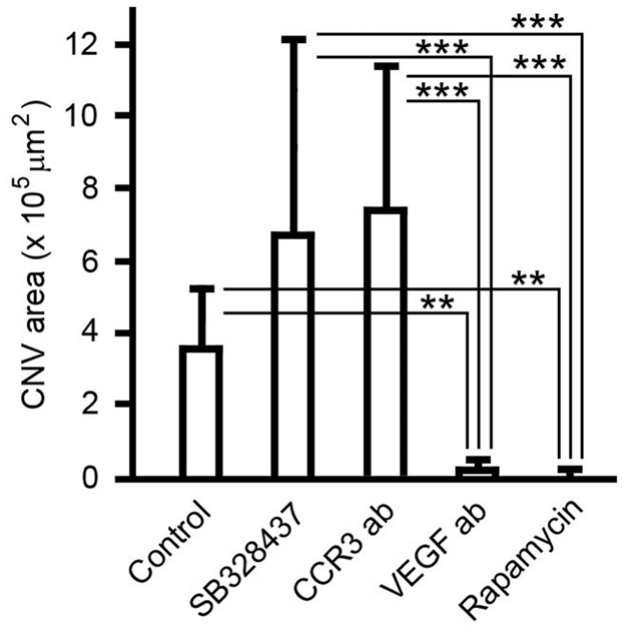
Quantification of CNV area in rats. The CNV areas were measured and calculated. No statistically significant difference was found between the control and either the SB328437 or the CCR3-ab group. On the other hand, the CNV areas of the VEGF-ab and the rapamycin group are significantly smaller than that of the control eyes (double asterisks, *P*<0.01) or eyes treated with SB328437 or CCR3-ab (triple asterisks, *P*<0.001).

Quantitatively, the CNV area is 3.63±1.64 (×10^5^ µm^2^, n = 13) in the control eyes. In the eyes treated with SB328437 or CCR3-ab, the CNV areas are 6.83±5.31 (n = 15) and 7.55±3.91 (n = 16), respectively (Fig. 5). In comparison, the CNV areas are 0.19±0.32 (n = 14) in the eyes treated with VEGF-ab, and 0.05±0.12 (n = 16) in rapamycin-treated eyes (Fig. 5). No statistically significant difference in CNV area was found between the control group and the group treated with SB328437 or CCR3-ab (Fig. 5). On the other hand, both VEGF-ab and rapamycin treatment significantly suppressed CNV development, as compared to the untreated group as well as the groups treated with SB 328437 or CCR3 antibodies (Fig. 5).

**Figure 6:**
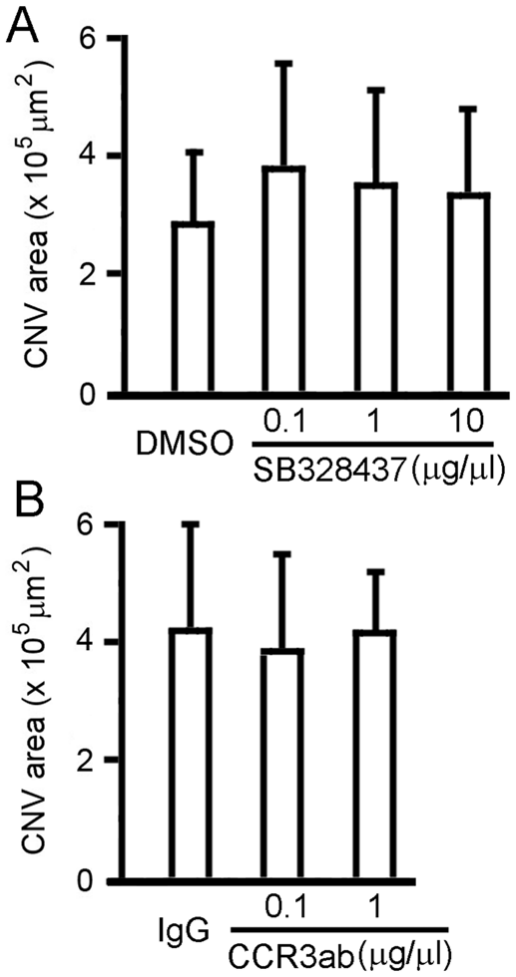
Dose range studies. Rat eyes were collected 12 days after injection of Matrigel with different doses of SB328437 or CCR3-ab. CNV was well developed in eyes injected with DMSO control (DMSO) and eyes treated with 0.1, 1, or 10 µg/µl SB328437 (A), as well as in eyes treated with isotype IgG control (IgG) and 0.1 and 1 µg/µl CCR3-ab (B). No statistically significant difference was found between the control eyes and treated eyes with either SB328437 or CCR3-ab of the doses used.

Dose range studies of the CCR3 inhibitors SB328437 and CCR3-ab showed no statistically significant difference between control eyes and treated eyes in either the SB328437 or the CCR3-ab group (Fig. 6). The CNV area is 2.90±1.24 (×10^5^ µm^2^, n = 5) in eyes injected with DMSO control (2.5% final concentration, equal to the amount of DMSO in Matrigel containing 10 µg/µl SB328437). In eyes treated with 0.1, 1, or 10 µg/µl SB328437, the CNV areas are 3.85±1.78 (n = 5), 3.56±1.61 (n = 6), and 3.39±1.45 (n = 7), respectively (Fig. 6A). The CNV area is 4.24±1.80 (n = 6) in the eyes injected with isotype IgG control (1 µg/µl). In eyes treated with 0.1 or 1 µg/µl CCR3-ab, the CNV areas are 3.79±1.75 (n = 6) and 4.23±1.41 (n = 5), respectively (Fig. 6B).

**Figure 7:**
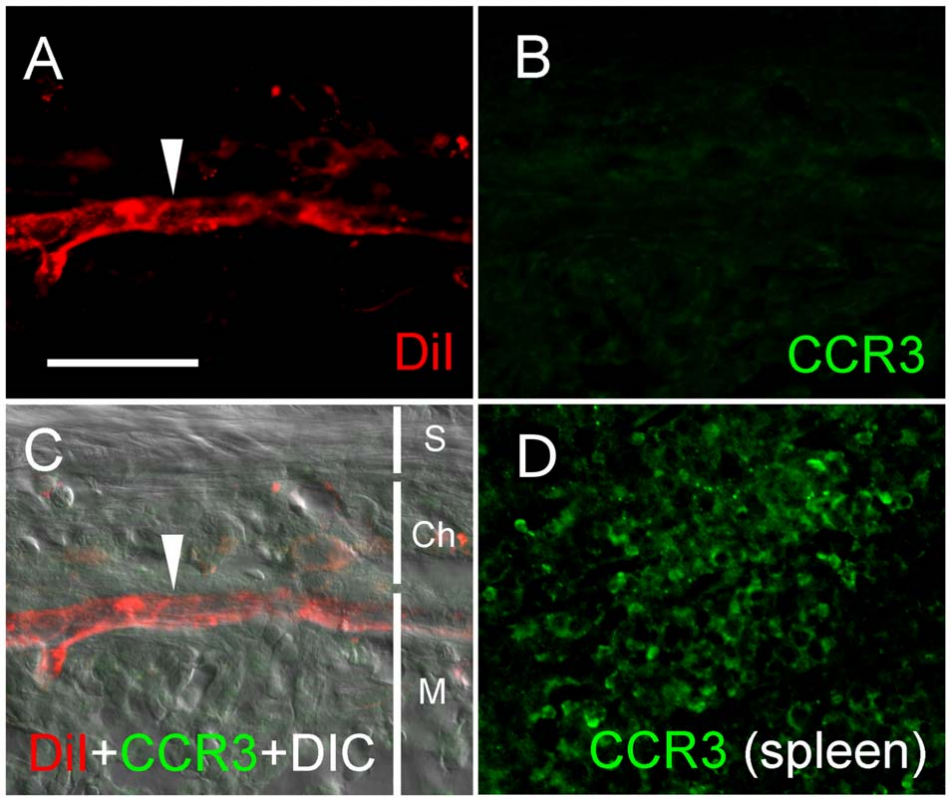
Expression of CCR3. Rat eyes were collected 12 days after injection of Matrigel. Cryosections were stained for CCR3 immunoreactivity. A well-developed new blood vessel is clearly present in the Matrigel injected area as revealed by DiI staining (A, arrowhead). No specific CCR3 immunoreactivity was detected in or around the blood vessel (B). A merged image of A, B, with a DIC image was presented in C (arrowhead indicates CNV). CCR3 positive staining is evident in a section of the spleen (D), which serves as a positive control. M, Matrigel area; Ch, choroid; S, sclera. Scale bar: 50 µm.

The expression of CCR3 in choroidal neovascular endothelial cells was examined by CCR3 immunostaining. No specific immunoreactivity of CCR3 was found in and around the CNV blood vessels (Fig. 7A, 7B and 7C) whereas CCR3 immnoreactivity is evident in the spleen (Fig. 7D), indicating that CCR3 is not specifically expressed in the CNV endothelial cells.

### CCR3 targeting and CNV development in mouse

**Figure 8:**
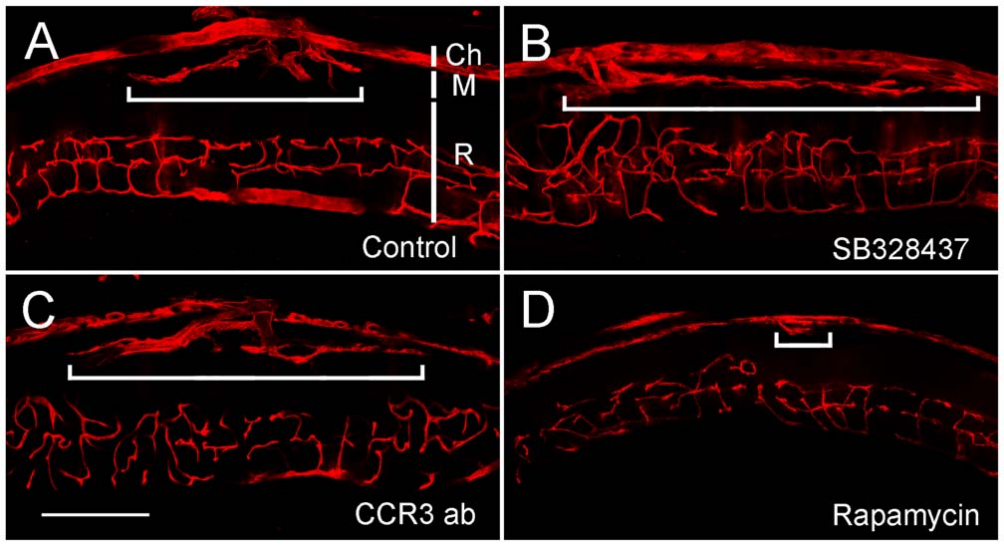
Effects of CCR3 or mTOR inhibition on CNV in mice. Eyes were injected with Matrigel alone or mixed with SB328437, CCR3-ab, or rapamycin, and collected 12 days later. Extensive CNV was seen in the control eyes (A) as well as the eyes treated with SB328437 (B), or CCR3-ab (C). In contrast, minimal CNV was found in eyes treated with rapamycin (D). The width of CNV is indicated by a horizontal bar in each section. The layers of the eye, including the choroids (Ch), the Matrigel area (M), and the retina (R), are indicated by vertical white bars in panel A. Scale bar: 200 µm.

**Figure 9:**
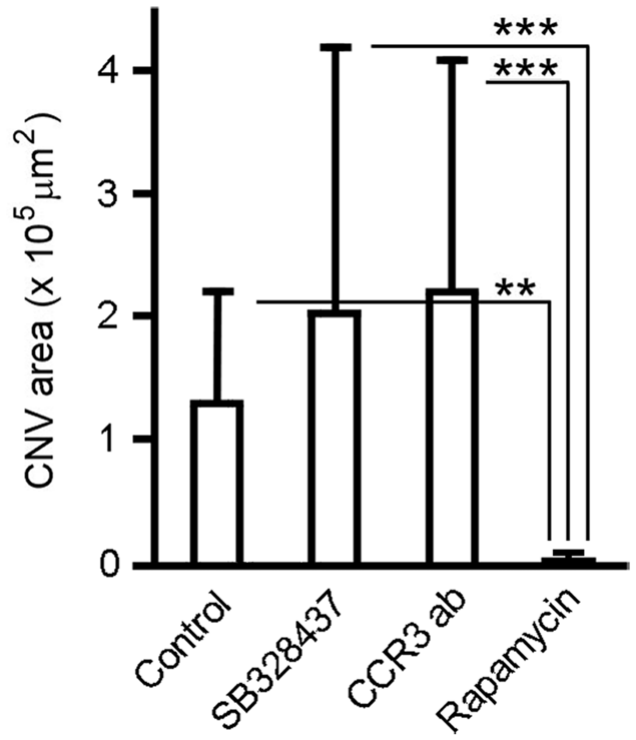
Quantification of CNV area in mice. The CNV areas were measured and calculated. No statistically significant difference was found between the control and the SB328437 or the CCR3-ab group. On the other hand, the CNV area in the rapamycin group is significantly smaller than that in the control eyes (double asterisks, *P*<0.01) or eyes treated with SB328437 or CCR3-ab (triple asterisks, *P*<0.001).

The failure of CCR3 targeting to inhibit CNV in rat prompted us to investigate whether CCR3 targeting could suppress CNV in mouse. Like in rat, subretinal injection of Matrigel induced extensive CNV in mouse (Fig. 8A). Again, no inhibition of CNV was seen in the eyes treated with either SB328437 or CCR3-ab (Fig. 8A and 8B). In contrast, significant CNV suppression was seen in rapamycin treated eyes. Among the 10 eyes treated with rapamycin, CNV is absent in 9 and only minimal CNV was found in one eye (Fig. 8D). Quantitatively, the CNV area is 1.29±0.92 (×10^5^ µm^2^, n = 14) in the control eyes, 2.03±2.1 (n = 15) in SB328437-treated eyes, and 2.2±1.89 (n = 22) in eyes treated with CCR3-ab (Fig. 9). In rapamycin-treated eyes, the CNV area is 0.008±0.024 (n = 10) (Fig. 9). No statistically significant difference is found in CNV areas between control eyes and eyes treated with either SB328437 or CCR3-ab (Fig. 9). In contrast, rapamycin treatment significantly suppressed CNV development, as compared to the control eyes and eyes treated with SB328437 or CCR3 antibodies (Fig. 9). These results are consistent with the data from rats and demonstrate that CCR3 is not critically involved in CNV development.

## Discussion

We have demonstrated that CCR3 targeting with either CCR3 neutralizing antibodies or high affinity CCR3 specific antagonist SB328437 has no inhibitory effect on CNV development in the subretinal Matrigel induced CNV in both rat and mouse. In addition, CCR3 expression was not detected in the CNV endothelial cells in our experiments. These results strongly argue against a critical role of CCR3 in CNV development and raise a question as to whether CCR3 is a valid therapeutic target for CNV. On the other hand, we have confirmed the role of VEGF-A in CNV development and that VEGF-A targeting is an effective approach to suppress CNV. Rapamycin, an mTOR inhibitor, is also efficacious in CNV inhibition.

CNV induction by subretinal Matrigel was first reported by Wen and colleagues in rat (Wen et al. *IOVS* 2002; 43:ARVO E-abstract 1297). In rabbits, subretinal Matrigel induces highly permeable neovescularization, as assessed by fluorescein leakage that lasted for weeks in some animals [26], consistent with the hyperpermeability of CNV found in the present work (Fig. 3). Subretinal Matrigel also induces RPE migration which eventually form a new RPE monolayer between photoreceptors and Matrigel and effectively turns the Matrigel location from subretinal to sub-RPE [22]. The Matrigel CNV model in rat has been characterized in detail in a recent report, showing that CNV in subretinal Matrigel progressively increases in size, accompanied by an increase in lesion size, a progressive infiltration of leukocytes and myofibroblasts, as well as deposition of collagen [8]. These features resemble the inflammatory reaction and fibrosis in wet AMD in patients. Thus, the Matrigel model closely mimics the CNV in wet AMD. VEGF-A neutralization and rapamycin treatment effectively suppressed CNV in the Matrigel model (reference [8] and present work), consistent with data from the laser-induced CNV model [6], [7], [21] and demonstrating the similarity between these two CNV models. Therefore, the discrepancy between our findings in the Matrigel CNV model and data reported by Takeda and colleagues in the laser-induced CNV model is unlikely due to the difference between the two CNV models. Our results that CCR3 targeting is ineffective in CNV inhibition in two species indicate that CCR3 is not critically involved in CNV development in general.

The results that VEGF-A neutralization effectively inhibited CNV development in both rat and mouse are consistent with many previous studies and confirm the critical role of VEGF-A in CNV development and maintenance. Thus, our present work provides additional support for VEGF-A targeting as a therapeutic strategy for wet AMD.

Rapamycin, originally identified as an antifungal agent [27], is a potent immunosuppressant [28], [29]. It forms a complex with the FK506 binding protein (FKBP-12) and then binds to mTOR (mammalian target of rapamycin) and interferes with its signaling [30]. As the central part of a complex intracellular pathway, mTOR is involved in the regulation of cell growth, metabolism, autophagy, and angiogenesis [31]. Studies have shown that rapamycin inhibits VEGF-A expression, VEGF receptor activation, and vascular endothelial cell proliferation [32], [33], [34], [35]. In addition, rapamycin inhibits HIF-1 expression, leading to inhibition of VEGF-A synthesis [35], [36]. Our results that rapamycin almost completely inhibited CNV development are consistent with a previous report that systemically administered rapamycin significantly suppressed laser-induced model of CNV in mouse [21], indicating that mTOR targeting is a potential treatment for CNV.

The report by Takeda and colleagues [20] was received with caution [37]. Our attempts to verify the role of CCR3 in CNV in the Matrigel model yield consistent negative results in two species, leading us to conclude that CCR3 is not critically involved in CNV development and maintenance and therefore CCR3 targeting is not a viable therapeutic approach for CNV. Our results, however, support the therapeutic strategies for CNV by VEGF-A and mTOR targeting.

## Materials and Methods

### Ethics Statement

All procedures involving animals adhered to Association for Research in Vision and Ophthalmology Statement for the Use of Animals in Ophthalmic and Vision Research and were approved by the Institutional Animal Care and Use Committee of University of Miami, Miller School of Medicine (07-232).

### Animals and subretinal injection of Matrigel

Adult Sprague Dawley rats and BALB/c mice (Charles River Laboratories, Wilmington, MA) were used in all experiments. CNV was induced by subretinal injection of Matrigel, as previously described [8], [22]. Briefly, animals were anesthetized with ketamine (40 mg/kg, i.p) and xylazine (6 mg/kg, i.p.). A 33G needle was used to make an insertion at the equator on the temporal side of an eye to reach the subretinal space. A blunt 33G needle attached to a 10-µl Hamilton micro-syringe was then introduced through the insertion to the subretinal space. Matrigel (growth factor reduced, BD Biosciences, Bedford, MA), diluted with phosphate buffered saline (PBS) or PBS containing CCR3, VEGF-A inhibitors, or rapamycin, at 3∶1 (75% gel), was slowly injected to form a bleb (volume of injection: 1.2 µl for rats, 0.8 µl for mice) (Fig. 1). The injecting needle was kept in place for 1–2 min for the gel to solidify before withdrawn. To synchronize the CNV development, an additional insertion at the center of the Matrigel injected area was made with a sharp 33G needle (Fig. 1).

### CCR3 and VEGF-A inhibition

CCR3 specific antagonist (S)-methyl-2-naphthoylamino-3-(4-nitrophenyl) propionate (SB328437, Calbiochem, San Diego, CA) or CCR3 neurtralizing antibodies (clone 83103, R & D Systems, Minneapolis, MN) was used for CCR3 inhibition. VEGF-A was blocked by VEGF-A neutralizing antibodies (R & D Systems). CCR3-ab or VEGF-ab was dissolved in PBS and then mixed with Matrigel before injection (1 µg/µl final concentration). SB 328437 or rapamycine (LC Laboratories, Woburn, MA) was dissolved in dimethyl sulfoxide (DMSO, Sigma-Aldrich, St. Louis, MO), diluted in PBS, and then mixed with Matrigel before injection.

### Visualization of blood vessels

Blood vessels were labeled with a DiI solution (1, 1′-Dioctadecyi-3, 3, 3′, 3′-tetramethylindocarbocyanine perchlorate, Sigma-Aldrich), as described [38]. Briefly, animals were killed by CO_2_ overdose and perfused with PBS, followed by the DiI solution. Tissues were fixed by subsequent perfusion with 4% paraformaldehyde (in 0.1 M phosphate buffer, pH 7.4). Eyecups were obtained by removing the anterior segment of the eye and post-fixed in the same fixative overnight, rinsed with PBS, and embedded in 5% agarose. Serial sections (100 µm) covering the entire Matrigel area were cut on a vibratome (VT1000S, Leica Microsystems, Bannockburn, IL), mounted on glass slides with 80% glycerol, and examined by confocal microscopy.

### Measurement of CNV

CNV area was calculated through the entire Matrigel area, as described [8]. The CNV area of a section (*C_i_*) was estimated by multiplying the “width” (*W_i_*), the maximal measurement of CNV along the sclera, by the “thickness” of the section *T_i_* (*C_i_ = T_i_ W_i_*). The height of CNV, the maximal distance between Bruch's membrane and the front edge of CNV, was not included since its variation was negligible. The thickness of each section (100 µm), *T_i_*, was the same for all sections as *T*. The entire CNV area of each eye (*C*) was calculated according to the equation: 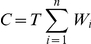



### Permeability of CNV

The permeability of CNV was examined with Evans Blue. Eyes of Sprague-Dawley rats were injected with Matrigel and 10 days later, Evans Blue (60 mg/kg in PBS) was given intravenously. Animals were killed 30 min after Evans Blue injection and perfused with PBS to clear the Evans Blue in the circulation system, followed by 4% paraformaldehyde (in 0.1 M phosphate buffer, pH 7.4) perfusion. Eyes were harvested and the anterior segments removed. Choroid-retina preparations were dissected, flat-mounted and examined by fluorescence microscopy.

### Immunostaining

Expression of CCR3 in the CNV endothelial cells was examined by immunostaining using antibodies against CCR3 (Santa Cruz Biotechnologies, Santa Cruz, CA) and visualized with Alexa fluor 647 conjugated secondary antibodies (Invitrogen, Carlsbad, CA). Eyecups were prepared as described above. Cryosections of 30 µm were cut and mounted on glass slides. Sections were rinsed in PBS and incubated in the primary anti-CCR3 antibodies (1∶50 dilution) at 4°C overnight, then in secondary antibodies (1∶250 dilution) at 4°C overnight. Sections were examined by confocal microscopy.

### Statistical Analysis

Results were analyzed by Kruskal-Wallis test followed by Dunn's test post hoc multiple analyses for comparisons between different groups, using InStat3 (GraphPad Software Inc., San Diego, CA). Data are expressed as mean±SD.
